# Genetic Mapping by Bulk Segregant Analysis in *Drosophila*: Experimental Design and Simulation-Based Inference

**DOI:** 10.1534/genetics.116.192484

**Published:** 2016-09-21

**Authors:** John E. Pool

**Affiliations:** Laboratory of Genetics, University of Wisconsin, Madison, Wisconsin 53706

**Keywords:** bulk segregant analysis, quantitative trait locus mapping, simulation, experimental design, *Drosophila*

## Abstract

Identifying the genomic regions that underlie complex phenotypic variation is a key challenge in modern biology. Many approaches to quantitative trait locus mapping in animal and plant species suffer from limited power and genomic resolution. Here, I investigate whether bulk segregant analysis (BSA), which has been successfully applied for yeast, may have utility in the genomic era for trait mapping in *Drosophila* (and other organisms that can be experimentally bred in similar numbers). I perform simulations to investigate the statistical signal of a quantitative trait locus (QTL) in a wide range of BSA and introgression mapping (IM) experiments. BSA consistently provides more accurate mapping signals than IM (in addition to allowing the mapping of multiple traits from the same experimental population). The performance of BSA and IM is maximized by having multiple independent crosses, more generations of interbreeding, larger numbers of breeding individuals, and greater genotyping effort, but is less affected by the proportion of individuals selected for phenotypic extreme pools. I also introduce a prototype analysis method for simulation-based inference for BSA mapping (SIBSAM). This method identifies significant QTL and estimates their genomic confidence intervals and relative effect sizes. Importantly, it also tests whether overlapping peaks should be considered as two distinct QTL. This approach will facilitate improved trait mapping in *Drosophila* and other species for which hundreds or thousands of offspring (but not millions) can be studied.

CONNECTING phenotypic diversity to the genetic variants that encode it is a fundamental challenge for modern biology. In evolutionary research, there is strong interest in revealing the genetic architecture of adaptive phenotypic change, including the number of causative genes and mutations, and their functional and population genetic properties. In molecular genetics, the mapping of phenotypic differences from natural or induced mutations has great utility for elucidating genetic pathways that underlie specific biological processes. In animal and plant breeding, localizing the genes underlying agronomically important trait variation can be a key step toward genetic improvement.

Especially in species that can be experimentally crossed, quantitative trait locus (QTL) mapping provides an important tool for identifying genomic regions that contain causative genetic variants underlying a trait difference. Often, the F_2_ or later offspring of a cross between phenotypically contrasting parental strains are genotyped, individually or in groups, to identify sections of the genome that were inherited nonrandomly with respect to the phenotype (often on the megabase scale). The simplest example of QTL analysis is F_2_ mapping, in which individual second generation offspring are phenotyped and genotyped. To achieve much genomic precision, however, this method requires the individual genotyping of a large number of F_2_ offspring. Preparing many genomic DNA libraries for next generation sequencing is often a time- and resource-intensive proposition, although progress has been made in this regard (Andolfatto *et al.* 2011).

Introgression mapping (IM) provides another alternative for QTL analysis. Here, following an initial cross between parental strains A and B, offspring of subsequent generations are repeatedly selected for strain A’s phenotype, but are back-crossed to strain B (Figure 1). To allow recessive variants to be selected, this selection and introgression can be performed in every second generation. The desired result is an introgression line that is largely similar to strain B across the genome, but that matches strain A at loci that were selected along with the phenotype. A notable modern example of this approach is described by Earley and Jones (2011), who introgressed a behavioral difference from *Drosophila simulans* into *D. sechellia*. Here, 30 F_2_ females were tested for *simulans*-like behavior, and a subset was then backcrossed to *D. sechellia*. After repeating this process for 15 generations, next-generation sequencing was used to identify genomic regions that introgressed with the trait from *D. simulans*.

**Figure 1:**
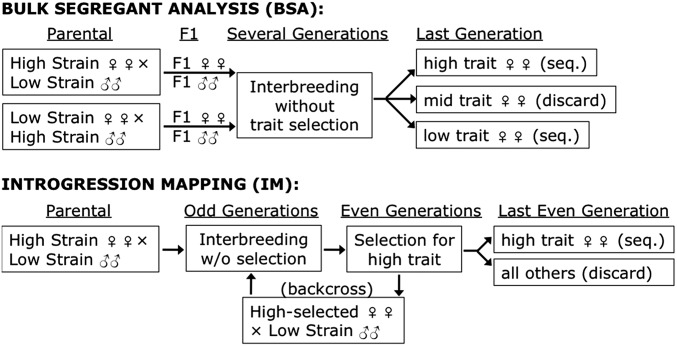
The investigated experimental designs for BSA and IM are illustrated. In BSA, offspring of reciprocal parental strain crosses are combined and allowed to breed without trait selection for a number of generations. Phenotyping occurs only in the final generation, and pools of individuals with the highest and lowest trait values are each sequenced. The IM framework investigated here involves trait selection and parental strain backcrossing every second generation (allowing recessive genotypes from the high parental strain to be expressed). In the last generation, one phenotypic extreme is sequenced and compared against the backcross parental strain genome.

In bulk segregant analysis (BSA), large numbers of progeny (from F_2_ or later generations) are sorted/selected by phenotype, then contrasting phenotypic pools of individuals are each genotyped (Figure 1) (Michelmore *et al.* 1991). Compared to IM, BSA may allow for a larger number of unique recombination events to be generated and sampled, which could yield sharper QTL peaks. Like IM, BSA does not require large numbers of offspring to be individually genotyped; instead, each phenotypic extreme can be sequenced as a single pool. BSA has been applied very successfully for selectable traits in yeast (*e.g.*, Ehrenreich *et al.* 2010; Magwene *et al.* 2011; Parts *et al.* 2011 facilitated by a small genome and the ease of generating millions of segregants. BSA has also seen diverse applications to trait mapping in multicellular organisms (*e.g.*, Michelmore *et al.* 1991; Wicks *et al.* 2001; Baird *et al.* 2008; Van Leeuwen *et al.* 2012; Haase *et al.* 2015), including *Drosophila* (Lai *et al.* 2007).

Here, I use simulations to examine the mapping signals of BSA and IM under a wide range of experimental parameters for the mapping of multigene traits. I find that BSA produces stronger and better-localized mapping signals for all studied experimental designs. The tradeoffs of effort and performance indicated by these results, along with the new simulation programs that produced them, will help researchers design more effective mapping experiments.

I also use this BSA simulation approach to devise a new QTL inference method. Existing BSA analysis methods effectively identify QTL from yeast data (*e.g.*, Magwene *et al.* 2011; Edwards and Gifford 2012). However, these methods do not allow the discrimination of two nearby QTL peaks *vs.* a single peak with noisy, ragged contours—an issue that may be more problematic for organisms in which many fewer segregants can be surveyed relative to yeast. These methods also do not estimate the relative strength of each QTL. The BSA inference method proposed here uses a multistep simulation process to (1) identify significant QTL and their genomic confidence intervals, (2) separate single- from multiple-linked QTL, and (3) provide a rough estimate of the effect sizes of the identified QTL. This method is validated using simulations in the present study and applied to data in an accompanying article (Bastide *et al.* 2016).

## Materials and Methods

### Preliminary simulations for BSA and IM

Simulation programs were written to assess the QTL signals of BSA and IM (software related to this article is available at https://github.com/JohnEPool/SIBSAM1). BSA simulation analyses focused on a summary statistic, “ancestry difference” (*a*_d_). For a given genetic marker locus or genomic window of sequence, *a*_d_ refers to the difference between the high and low phenotypic pools in the proportion of ancestry from the parental strain with the higher phenotypic value. For example, if the high phenotypic pool is estimated to have 60% of its ancestry from this parental strain at a particular locus, and the low phenotypic pool 40%, then *a*_d_ = 0.6 − 0.4 = 0.2. For IM, the proportion of ancestry in the mapping population from the nonbackcross parental strain (*a*_p_) was evaluated. This quantity may approach zero for noncausative loci after many generations of backcrossing to the other parental strain. For each statistic, I examined how often the tallest local QTL peak was observed within 0.5 cM of the true simulated target locus and the average (median) distance between the QTL peak and the target locus.

The BSA and IM simulators are largely similar. These programs track parental strain ancestry along the chromosomes of each individual in the mapping population, from the F_1_ generation until the end of the experiment. A Poisson-distributed number of recombination events happen each generation, with the expected number for each chromosome being its length in morgans (interference is not modeled). To focus on the case of *Drosophila*, chromosomes X, 2, and 3 were explicitly simulated, and no recombination was allowed in males. A total of 5000 markers/windows were simulated on each chromosome. In the BSA simulation, a specified number of individuals exist in each new generation, and each one draws random parents from the previous generation, with no phenotypic selection until the last generation. In the IM simulations, individuals were subject to phenotypic selection in every second generation (allowing recessive alleles from both parental strains to be expressed), and the selected individuals were backcrossed to one of the parental strains.

Phenotypes for each individual were modeled based on genotypes and random variance (the latter may stem from environmental effects, measurement error, or other causes). For most of these preliminary simulations, the same number of equal-effect loci were simulated on each chromosome arm (X, 2L, 2R, 3L, and 3R). Random variance was added by modifying each individual’s phenotypic value by a normally distributed random effect with mean 0 and SD equal to the average trait value. For example, if each of the five arms holds a single QTL that adds 1 to a diploid individual’s phenotypic value for each allele inherited from the high parental strain, the range of genetic contributions could range from 0 to 10, with a mean of 5, and the SD for environmental variance would also be 5. Phenotypic selection was then based on choosing a defined quantile (*q*) of individuals from the mapping population with the highest and the lowest phenotypic values.

For BSA, phenotypic selection happens only at the end of the experiment, followed by sequencing/genotyping of both high and low phenotypic pools. For IM, the last batch of selected individuals is sequenced and compared against the parental strains. The simulations model “depth” of sequencing coverage (or genotype sampling), drawing an appropriate number of random ancestry-informative reads from the selected pool of individuals for each window/marker. The proportion of ancestry from each parental strain is then calculated, and thus depends on both the sampling of individuals and the sampling of sequence reads.

To facilitate consistent analysis, QTL in these preliminary simulations were spaced uniformly and each was assigned a specific analysis zone along the chromosome. For example, if the X chromosome had five QTL, they would be placed at relative positions 0.1, 0.3, 0.5, 0.7, and 0.9 (representing the chromosome as a 0-to-1 interval). Their zones of analysis would then be 0 to 0.2, 0.2 to 0.4, and so on. The assessment of QTL signal strength and precision was based on the location within its zone of the highest QTL peak (*i.e.*, the maximum *a*_d_ or *a*_p_), relative to the true QTL position.

Most simulation analyses assumed that each mapping experiment would be analyzed separately. However, I also investigated cases where multiple independent mapping populations were constructed from parental strains sharing the same causative genetic differences. Here, *a*_d_ or *a*_p_ for each window was summed across replicated mapping populations.

For a wide variety of experimental parameter combinations, 1000 independent replicates were simulated and analyzed, and statistical performance was compared between these scenarios to aid in the optimization of experimental design.

### Simulation-based inference of QTL from BSA: Overview

Preliminary empirical BSA data from the Pool laboratory indicated the need for a QTL inference method capable of dealing with neighboring QTL that have wide, overlapping statistical signals. Such scenarios are difficult to account for in most analysis approaches, but the simulation framework described above offers a potentially flexible foundation for QTL inference. I therefore developed a method of simulation-based inference for bulk segregant analysis mapping (SIBSAM). SIBSAM uses BSA simulations analogous to those described above, with null model simulations yielding *P*-values for each QTL peak, and an approximate Bayesian approach providing estimates of QTL strength and genomic confidence intervals. An important feature of SIBSAM is the ability to distinguish individual QTL among clusters of linked causative loci.

**Figure 2:**
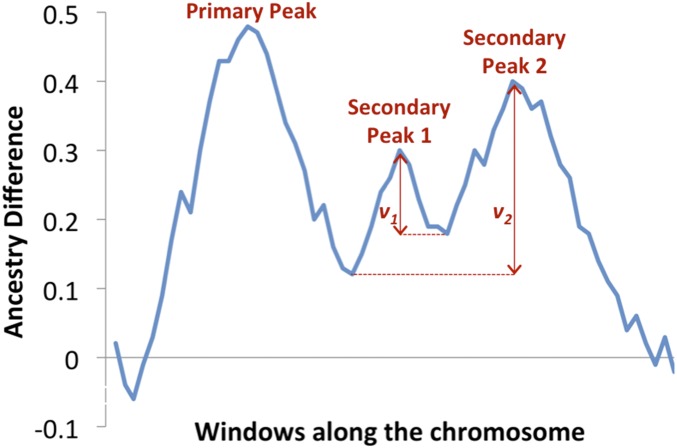
Definitions of primary and secondary peaks, along with secondary deviation, used by SIBSAM are illustrated here. Among a group of contiguous windows with smoothed *a*_d_ values >0, the primary peak is defined by the window with the highest value. Secondary peaks represent other local maxima, and their significance is judged based on secondary deviation (*v*). Secondary deviation is determined by the difference in *a*_d_ between the secondary peak’s maximum value and the minimum value between that peak and the primary peak (or a taller secondary peak, whichever minimum is greater).

Throughout the SIBSAM pipeline, the distinction between primary QTL peaks and secondary QTL peaks is relevant. A primary QTL peak is defined based on the highest value of *a*_d_ across a continuous interval in which this statistic remains >0 (which is the null value expected in the absence of causative loci). A secondary QTL peak within that same interval has a lower height than its associated primary peak. An important quantity in assessing the significance of a secondary peak is its “secondary deviation” (*v*), defined as the difference between secondary peak height and the minimum *a*_d_ value between the primary and secondary peaks (Figure 2). Multiple secondary peaks may be associated with the same primary peak, impacting the calculation of *v*, as discussed below.

**Figure 3:**
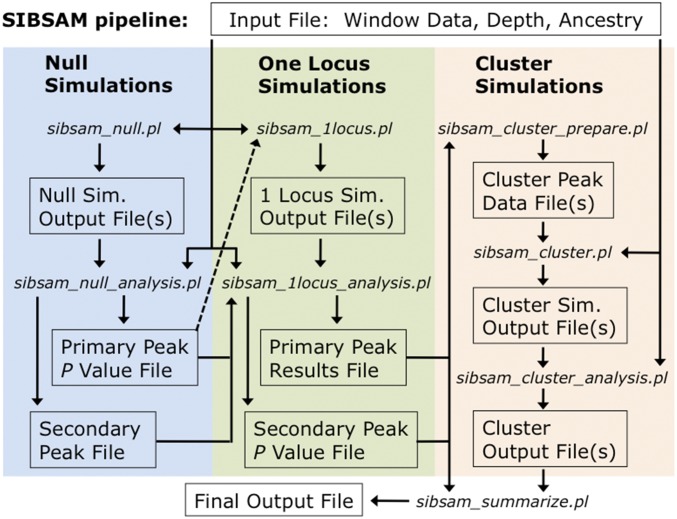
A flow chart illustrating the SIBSAM analysis pipeline. A single input file contains physical and genetic map positions of window boundaries for all chromosomes, along with ancestry difference values and informative depth (the number of reads within information about parental strain ancestry) for each window. Null simulations with no true QTL are used to identify significant primary peaks in the empirical data. Simulations with one QTL (matching a primary peak location) are then used to estimate confidence intervals for primary peak effect size and genomic location, while also identifying significant secondary peaks. For any primary peak with significant secondary peaks, cluster simulations are conducted with QTL at each peak’s location, to generate final confidence intervals for effect size and genomic location. These analyses are summarized into a single output file containing all relevant inferences for each significant peak.

A schematic of the SIBSAM pipeline is illustrated in Figure 3. First, primary and secondary peaks of *a*_d_ are identified from the empirical data. To determine which primary peaks are unexpected in the absence of true QTL, null simulations are conducted in which phenotypes are determined by nongenetic factors only. *P*-values can then be obtained for each primary peak. Next, simulations with a single causative QTL are conducted. Based on a rejection sampling approach, estimates of the strength and genomic confidence intervals of each significant primary peak are obtained, along with a *P*-value for each secondary peak. Lastly, simulations involving a cluster of linked QTL are conducted, reflecting a primary peak and its associated secondary peak(s). This phase allows for the refinement of strength estimates and genomic confidence intervals for each peak in the cluster.

All of the above simulations operate with user-defined windows of variable centimorgan length. These windows could also be viewed as markers separated by various centimorgan distances, but this article’s terminology mainly assumes that QTL mapping data come from the full resequencing of mapping population genomes. In the examples presented here, the window base pair spans were based on *D. melanogaster* polymorphism data (Lack *et al.* 2015) and centimorgan distances were calculated from empirical recombination rate estimates (Comeron *et al.* 2012). Windows were defined to each contain 200 nonsingleton variable sites from the Zambia–Siavonga population sample. The user can also define the “informative depth” for each window in each phenotypic pool. This quantity refers to the number of sequence reads that contain information about parental strain ancestry. The simulator will draw a corresponding number of alleles at this window for ancestry proportion calculations.

### SIBSAM identification of primary and secondary peaks from empirical data

Primary and secondary peaks of *a*_d_ are identified from data based on preliminary thresholds for primary peak height and secondary peak deviation (*a*_dt_ and *v*_t_, respectively), plus an optional smoothing step. The two thresholds should represent values low enough that no shorter peak would be statistically significant (the default value for both is 0.1). The smoothing enabled here is a simple weighted average. On each side of the focal window, *m* flanking windows are included (the default used here is *m* = 4). The focal window receives a weight of *m* + 1, the adjacent window on each side receives a weight of *m*, the next windows receive a weight of *m* − 1, and so on until the *m*th window to each side receives a weight of 1. Alternative smoothing schemes are not a focus of this study; the optimal strategy should depend on the data being analyzed. Empirical and simulated *a*_d_ values must be smoothed using the same procedure.

Primary peak identification is straightforward: the highest value of *a*_d_ in a continuous block of windows with *a*_d_ > 0, conditional on the peak value of *a*_d_ exceeding *a*_dt_. To identify secondary peaks, local minima and maxima of *a*_d_ moving away from the primary peak are noted. A recovery, beyond *v*_t_, from the low point since the last peak signifies a new secondary peak. When *a*_d_ drops more than *v*_t_ below this secondary peak’s maximum value, this peak ends and its maximum value and associated window position are noted. Statistical significance of these primary and secondary peaks, along with their confidence intervals and relative strengths, will be assessed in subsequent stages of this pipeline.

### SIBSAM identification of significant primary peaks

The false positive probability (*P*) for each primary peak is estimated by comparing empirical *a*_d_ peak heights against simulations under the null hypothesis of no true QTL, in which all phenotypic variance in the mapping population is random with respect to genotype. All primary peaks exceeding *a*_dt_ from each simulation replicate are noted. The enrichment (*e*) of peaks equal to or greater than a given peak’s height in the real data are given by the ratio of the frequency of peaks of this height in the real data relative to the simulated data. If there is an enrichment (*e* > 1), an estimate of the proportion of real peaks of this height representing false positives is then given by 1/*e*. For example, if *a*_d_ peaks of at least 0.2 in height are three times more common in the empirical data than in null simulations, then on average one out of three such empirical peaks can be explained by the expected false positive rate. Primary peaks with an estimated *P* less than some threshold (by default, 0.05) are carried forward for subsequent analysis.

### SIBSAM inferences from single QTL simulations

Genomic simulations with a single QTL are used to estimate the genomic confidence intervals and strength of each significant primary peak, along with a *P*-value for each secondary peak. Single QTL simulations are performed with fixed genomic positions corresponding to the window with the peak maximum *a*_d_ for each QTL, thus conserving local window patterns of depth and centimorgan distance. For a given set of simulated genomes from the mapping population (preselection), a random QTL effect size is drawn from an uninformative prior distribution (ranging from 0 to 1). Such a QTL is then separately simulated at each position corresponding to an empirical primary peak, with phenotype simulation and read sampling performed separately in each case. The simulated ancestries are reused for each separate QTL simulation as a time-saving efficiency.

The simulated QTL strength, *s*, ranging from 0 to 1, is the estimated proportion of variance that a QTL explains among the mapping population individuals. In these single locus simulations, all other phenotypic contributions are modeled as random variance, which here is intended to encompass the effects of unlinked QTL in addition to nongenetic effects on phenotypic measurements. The amount of random variance simulated is fixed to approximate the variance contributed by a codominant locus in which each allele adds 1 to the phenotypic score. This effect was implemented by obtaining Gaussian random values with mean 0 and SD 1, and then multiplying each value by $\sqrt{0.5}$ to obtain the random variance effect on each individual’s phenotypic score. The simulated effect size of each QTL, *f*, describes the quantity that each allele of this locus (inherited from the high parental strain) adds to an individual’s phenotypic score. Since random effects correspond to the variance contributed by a locus with *f* = 1, the proportion of variance contributed by a single QTL (*s*) is equal to *f*/(1 + *f*). And correspondingly, a single QTL intended to have strength *s* is simulated with an effect size *f* = *s*/(1 + *f*).

A simple approximate Bayesian framework, using rejection sampling based on observed *a*_d_ peak height, is used to estimate *s* and its confidence interval, along with a confidence interval for genomic location. For each simulated replicate, the simulated strength is recorded, along with each QTL’s maximum *a*_d_ height, peak window location, and maximum secondary deviation. To analyze the one locus simulation data for each primary peak, a rejection sampling approach is used to identify simulation replicates in which maximum *a*_d_ falls within a specified tolerance (default 0.025) of the empirical peak’s maximum *a*_d_. For each accepted simulation replicate, the strength of the simulated locus goes into the posterior distribution for the empirical QTL’s strength (from which strength values corresponding to the 0.05, 0.5, and 0.95 quantiles are returned). A genomic confidence interval is similarly obtained by examining the far left and far right quantiles for the simulated peak locations resulting from a QTL simulated at the empirical peak location. This assumes a certain transitivity. SIBSAM simulates QTL with fixed positions and observes how far away the maximum *a*_d_ falls in these simulations. In the empirical data, one observes the location of the maximum *a*_d_, and would like to know how far from this window the true QTL might be. Thus, the method assumes the distances from true QTL to maximum *a*_d_ in the simulated data are a good proxy for the distances between maximum *a*_d_ and true QTL in the empirical data.

Lastly, the secondary deviations from each accepted simulation enable *P*-values to be calculated for each of the empirical primary peak’s associated secondary peaks. If more than one secondary peak is present on the same side of the primary peak in the empirical data, the tallest secondary peak is tested first, and its *v* is based on the difference between its height and the lowest *a*_d_ value between itself and the primary peak (even if other secondary peaks exist between this peak and valley; Figure 2). For a shorter secondary peak between a primary peak and a taller secondary peak, *v* would be defined as the difference between its height and the higher of the valleys on either side of it. Giving taller peaks this priority avoids the situation of a shorter secondary peak being deemed significant and a taller peak beyond it missing this threshold (as might occur if secondary peaks were simply evaluated sequentially by position). After such adjustments, each secondary peak deviation in the empirical data associated with this primary peak is compared to the distribution of *v* from accepted simulations only. The proportion of simulations with a *v* greater than observed for a given empirical secondary peak estimates the *P*-value for that peak (*i.e.*, the probability of getting a secondary deviation this extreme when the true model is a single QTL of the observed magnitude). Rejection sampling based on primary peak height thus allows the approximation of secondary peak *P*-values in the absence of precise knowledge of the primary QTL’s strength, which here represents a “nuisance parameter” that will impact the expected width of the QTL interval, and hence the distribution of *v* expected from it.

### SIBSAM inferences from QTL cluster simulations

In cases where an empirical primary peak is accompanied by one or more statistically significant secondary peaks, the strengths and confidence intervals of all peaks in this “QTL cluster” are best approximated from simulations that include each member QTL. For example, a pair of nearby QTL may each add to the *a*_d_ peak height of the other, leading to overestimates of effect size. Therefore, multi-QTL simulations are conducted separately for each QTL cluster inferred from the empirical data. For simplicity, the window position of each simulated QTL is fixed according to the windows showing maximum *a*_d_ for each significant peak in the empirical cluster. To examine each QTL separately, each is assigned an analysis zone with boundaries corresponding to the empirical valleys (local minima) between peaks. Moving away from the outer peaks in the cluster, this analysis zone is bounded only by the ends of the chromosome.

For each cluster simulation replicate, a random strength value is first drawn for the full cluster (representing the cumulative proportion of phenotypic variance explained by the QTL in this cluster). That cluster strength is randomly apportioned among the QTL, and each peak’s strength is then translated into the simulated effect size as described above.

Posterior estimates for each QTL’s strength and genomic location are obtained from an approximate Bayesian procedure similar to that described above for the analysis of single QTL simulations. But here, a cluster simulation replicate is accepted only if the local maximum *a*_d_ in every QTL’s analysis zone falls within a tolerance of the corresponding empirical peak heights. Since matching multiple QTL heights may entail lower acceptance rates, it could be necessary to use a slightly higher tolerance value to accrue enough accepted simulations (default *a*_d_ tolerance 0.05). This or any other simulation step in SIBSAM can be parallelized to increase the number of replicates, followed by joint analysis of multiple simulation output files (Figure 3).

The estimated strength of each peak in cluster, along with confidence intervals of strength and genomic position, are obtained from a similar rejection process as described for the one locus simulations (based on the distribution of strength values and peak locations for that peak among the accepted simulations). Thus, the cluster QTL simulations provide estimates of effect size and genomic confidence intervals for all significant secondary peaks. They also replace prior estimates of these quantities for the associated primary peaks, since cluster estimates that account for the effects of linked QTL should be more accurate.

In summary, SIBSAM utilizes heuristic statistics based on ancestry differences (primary peak height and secondary deviation) in a null simulation framework to test the significance of QTL peaks and uses peak heights in a simple approximate Bayesian framework to estimate the strength and genomic location of both individual QTL and QTL in linked clusters. The final SIBSAM output file contains, for each significant primary and secondary peak, its *P*-value, the genomic coordinates of the peak window, the confidence interval for the QTL’s genomic location, and the point estimate and confidence interval for QTL strength. Information such as *P*-values for nonsignificant peaks can be found in the intermediate files produced at different stages of the SIBSAM pipeline (Figure 3).

### Simulations testing the performance of SIBSAM

Simulation testing of SIBSAM was performed to test its QTL detection power under different scenarios and to confirm that estimates and confidence intervals of genomic location and QTL strength were performing in line with expectations. Although a nearly infinite range of scenarios could potentially be investigated, I focused on experimental parameters relevant to our current empirical applications in *Drosophila* (*e.g.*, Bastide *et al.* 2016), in which 1200 individuals interbreed for 16 generations, and 10% phenotypic tails are selected for sequencing. Test simulations sampled 1000 informative reads for each window for each phenotypic pool, which is about half the median depth per window from current empirical applications (*e.g.*, Bastide *et al.* 2016). Windows were designed to each contain 200 nonsingleton variable sites in the Zambia–Siavonga population genomic data described by Lack *et al.* (2015). These 14,107 windows had a median length of 6.8 kb.

Simulations with one genuine QTL were performed with varying locus strengths (*s* = 0.05, 0.1, 0.15, 0.2, 0.25, 0.33, and 0.5). These initial test simulations used fixed genomic positions corresponding to the locations of *Drosophila* pigmentation genes *tan* (on the X chromosome) and *ebony* (on arm 3R). Additional 3R scenarios with *s* = 0.2 investigated the consequences of the remaining variance being due to unlinked QTL (one with *s* = 0.8 or else four others with *s* = 0.2) instead of random Gaussian variance. Comparing each test replicate against SIBSAM null simulations revealed the true positive rate for QTL detection. Running the test replicates through the SIBSAM, one locus simulation analysis indicated the frequency at which secondary QTL were falsely inferred, along with allowing the inferred distributions of QTL strength and genomic location to be compared against known true values.

Additional simulations were conducted (focusing on the 3R location) to investigate SIBSAM’s performance in the presence of two linked QTL. Scenarios with symmetric QTL strength (*s* = 0.15 or 0.3) and asymmetric QTL strength (*s* = 0.15 and 0.3) were investigated. The distance between the two QTL was varied at 2.5, 5, 10, and 25 cM. The test replicates were then evaluated with SIBSAM to (1) test the power to detect one or both QTL, (2) test the rate of falsely detecting three or more QTL, (3) evaluate the performance of QTL localization, and (4) evaluate the performance of QTL size estimation.

### Data availability

The authors state that all data necessary for confirming the conclusions presented in the article are represented fully within the article. Source code is available from https://github.com/JohnEPool/SIBSAM1.

## Results

### Initial simulation study of BSA and IM

Simulations were performed to examine the properties of QTL signals under BSA and IM approaches. Importantly, these exploratory simulations are not connected to any formal QTL inference. Instead, they focus on the performance of summary statistics related to the signature of a QTL. For BSA, I examine ancestry difference (*a*_d_), the difference between high and low phenotypic pools in the proportion of ancestry sampled from the parental strain with the higher phenotypic value (at a particular genomic locus). For IM, I examine ancestry proportion (*a*_p_), the proportion of the mapping population’s ancestry that derives from the nonbackcross parental strain. Rather than focusing on the raw values of these statistics, I assess the performance of BSA and IM by examining the genetic distance between a true simulated QTL and the “QTL peak” (the maximum value of *a*_d_ or *a*_p_ in this part of the genome).

The above approach allows a wider range of scenarios to be examined than would be computationally feasible under the full SIBSAM inference process. Beyond a tentative comparison of the genomic precision of BSA *vs.* IM, an important goal here is to optimize critical experimental parameters to improve the outcomes of future trait mapping studies.

As a point of reference, these simulations began with a “default” scenario in which 600 individuals were bred each generation, for 10 total generations, phenotypic selection retained the 20% most extreme individuals in each direction, and each window/locus had a sequencing depth of 300. Individual parameters were then varied, alone or in combination, and the accuracy of the *a*_d_ or *a*_p_ signal was examined.

**Figure 4:**
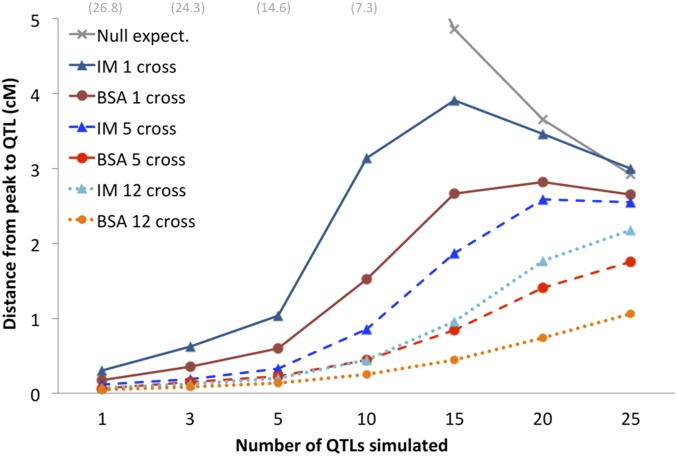
Results are shown for exploratory BSA and IM simulations with varying numbers of QTL and numbers of jointly analyzed independent crosses. As a proxy for method performance, the median centimorgan distance between the true QTL and the statistic maximum (of *a*_d_ for BSA or *a*_p_ for IM) is shown. The null expectation for a randomly located peak within a QTL’s analysis window is also shown (gray). These results indicate: (1) the increasing challenge of more polygenic scenarios for all approaches, (2) a general advantage of BSA over IM, and (3) the utility of combining data from independent crosses that all share a given QTL in common.

First, performance was examined when tandemly varying the number of QTL and the number of independent crosses. Within each simulation case, all QTL were of equal magnitude and explained 5/6 of total phenotypic variance. Independent crosses could represent cross replicates using the same parental strains, or distinct pairs of parental strains from the same populations that may share some QTL in common. Here, multicross simulations assumed that all crosses shared a given QTL difference between them, and *a*_d_ or *a*_p_ were added between crosses for each genomic window to test whether a more precise localization emerged from this joint signal. Three primary themes emerged from this analysis. First, BSA outperformed IM for any given combination of crosses and loci (Figure 4). Second, combining data from multiple crosses had a markedly positive effect on the accuracy of these ancestry signals. Third, performance showed a predictable decline for more/weaker QTL. Still, cases with multiple crosses managed relatively stronger performance for more polygenic scenarios (Figure 4), particularly in the case of BSA. For simplicity, the remaining simulations below will focus on a single cross replicate and a scenario with five QTL.

**Figure 5:**
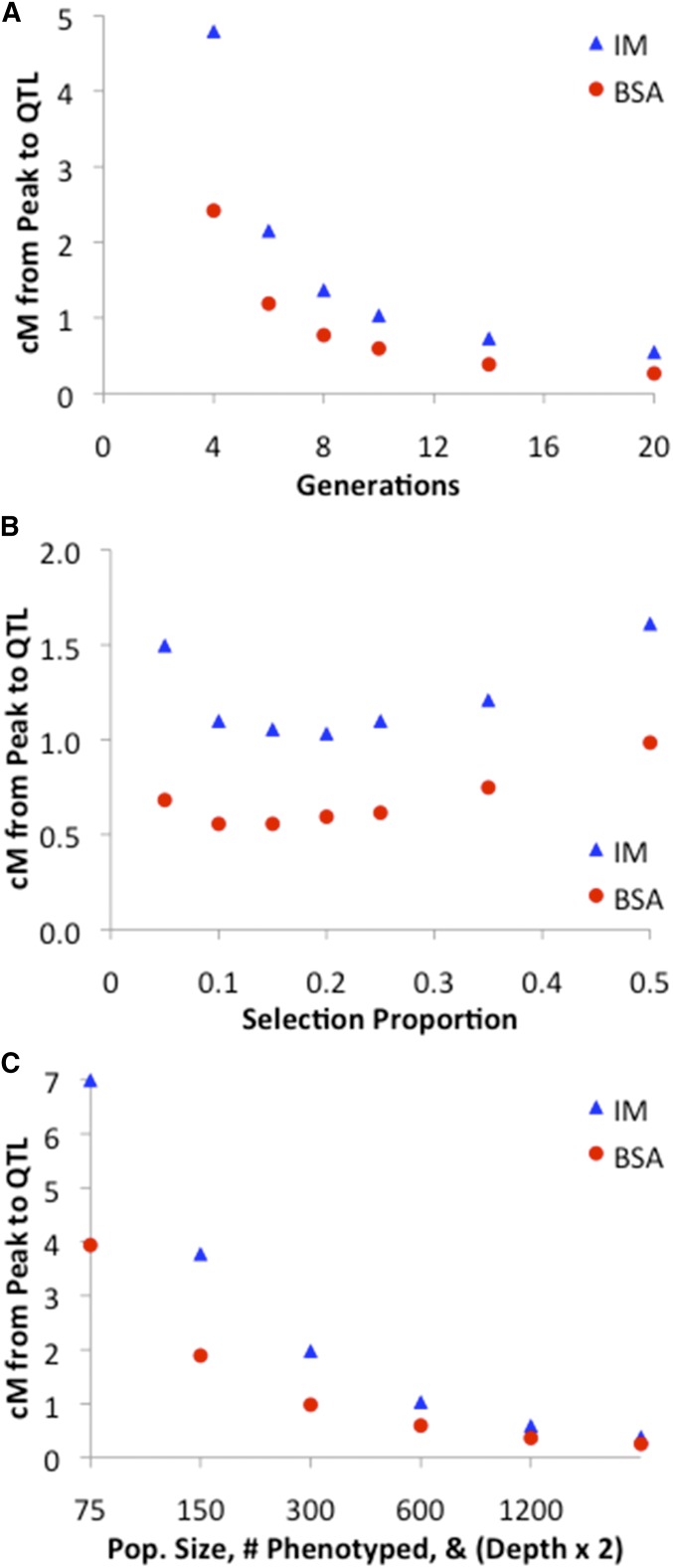
The results of exploratory BSA and IM simulations are shown in which one or more experimental variables were manipulated. (A) Increasing the total number of generations in the experiment reduces the median centimorgan distance between the true QTL and the observed peak. (B) A broad optimal range of selection proportion exists for the focal BSA and IM scenarios. (C) Scaling up the experimental population size (and hence the number of phenotyped individuals), along with the sequencing depth, leads to improved statistical performance.

The number of generations before genotyping/sequencing was also varied. Strong performance improvement was observed by increasing the number of generations to 8 or 10, with further increases yielding ongoing but diminishing improvements (Figure 5A). Additional generations allow more recombination between parental genetic backgrounds, which should lead to sharper QTL peaks.

Past results indicate that selecting only the most extreme individuals is not optimal for BSA (Magwene *et al.* 2011). Concordantly, for the focal simulation scenario studied here, optimum bulk proportions were ∼10–15% for each BSA pool, and 20% for the single IM pool (Figure 5B). These results appear to reflect a balance between enriching for causative genotypes (favoring fewer individuals) and minimizing the effects of random sampling variance (favoring more individuals). Thus, both BSA and IM studies may benefit from selecting significant numbers of individuals, which should help to maximize the diversity of recombination breakpoints represented in the final data.

**Figure 6:**
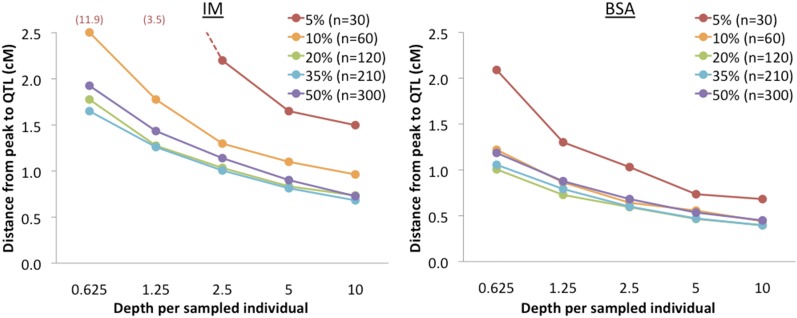
Outcomes of exploratory BSA and IM simulations with variable sequencing depth are shown. To more clearly illustrate the influence of depth on sampling variance, depth is plotted in terms of the average number of reads for each individual in a phenotypically selected pool. From a group of 600 phenotyped individuals, results for a series of selection proportions are illustrated. Results illustrate the advantage of increased sequencing depth, with some diminishing returns.

Related to the issue of sampling variance are parameters such as the number of individuals present in each generation and the number of genotypes sampled in the data (*e.g.*, sequencing depth). When simulations jointly scaled up the number of individuals present in each generation, the number sampled for sequencing, and the sequencing depth, performance improved considerably (Figure 5C). The number of individuals sampled in the final generation made a particular difference, at least if depth was scaled up linearly (Supplemental Material, Figure S1). Increasing sequence depth consistently led to better performance (via a reduction in sampling variance), although with some diminishing returns (Figure 6).

Simulations also considered the interaction between selection proportion and population size. The optimal selection proportion (*s*) tends to scale inversely with population size (*N*). For BSA population sizes between 100 and 2400, there was a relative stability in the optimal number of sampled individuals for sequencing (*Ns*), with this quantity ranging only from 35 to 60 (Table S1). In line with the findings of Magwene *et al.* (2011), this result suggests that reducing sampling variance is of primary importance, whereas enriching for the most phenotypically extreme individuals is a secondary priority.

### Simulation testing of the SIBSAM pipeline

As elaborated in the *Materials and Methods* section, I developed a prototype method for simulation-based inference for BSA mapping (SIBSAM). The flexibility of this simulation-driven pipeline allows a range of inferences, including for challenging cases in which two or more QTL are part of the same complex peak (Figure 2). The goals of SIBSAM include assessing the significance of peaks, and estimating the strength and genomic confidence interval of significant QTL. The performance of SIBSAM was assessed via a series of test simulations with one or more QTL. While a vast range of QTL and experimental scenarios could potentially be examined, I focus here on parameters relevant to ongoing empirical work in *Drosophila* (Bastide *et al.* 2016). The BSA experimental design simulated here went for 16 generations, with 1200 individuals in each generation, with 600 females phenotyped in the last generation with 10% pools selected, and 1000 informative sequence reads for each genomic window.

**Figure 7:**
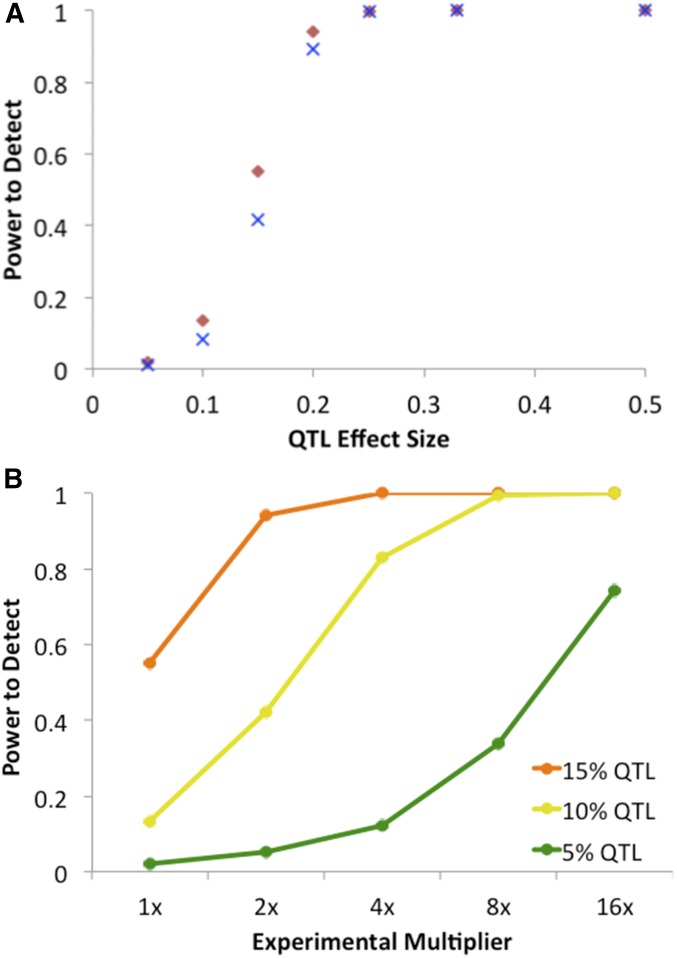
(A) Results of one locus test simulations assessing the power of the SIBSAM pipeline to detect a QTL on the autosomes (red diamond) or the X chromosome (blue X). The scenario investigated here involves a population of 1200 individuals with 600 phenotyped after 16 generations and 10% retained in each phenotypic pool, and with a depth of 1000 informative sequencing reads per window. This scenario showed intermediate power for a QTL explaining 15% of phenotypic variance in the experimental population, with low/high power below/above that mark. (B) The ability of a larger experiment to boost detection power for weaker QTL was investigated. The mapping population size, number of individuals phenotyped, and sequencing depth was multiplied (compared to the numbers given above) as shown on the *x*-axis. In a sufficiently large experiment, the power to detect QTL explaining 10% or even 5% of phenotypic variance was considerably increased.

For the above scenario, SIBSAM’s QTL detection power went from weak for a QTL explaining 10% of the experimental population’s phenotypic variance (with the remainder due to random environmental or measurement variance) to strong for a 20% QTL, with intermediate power for 15% QTL (Figure 7A). The low power for weaker QTL could indicate that a larger mapping experiment is needed to detect them. A second set of test simulations was conducted in which mapping population size, the number of phenotyped individuals, and the sequencing depth were each multiplied by either 2, 4, 8, or 16, relative to the focal scenario. For example, since the focal scenario involves selecting on the phenotypes of 600 individuals, the 16x scenario involves 9600 individuals. These scaled-up experiments resulted in higher power to detect QTL of strength 5, 10, or 15% (Figure 7B). Scaling up by a factor of 4 resulted in 83% power to detect a 10% QTL, while a multiplier of 16 was necessary to attain 74% power for a 5% QTL. Thus, larger mapping experiments substantially improve the prospects for detecting weaker QTL.

**Figure 8:**
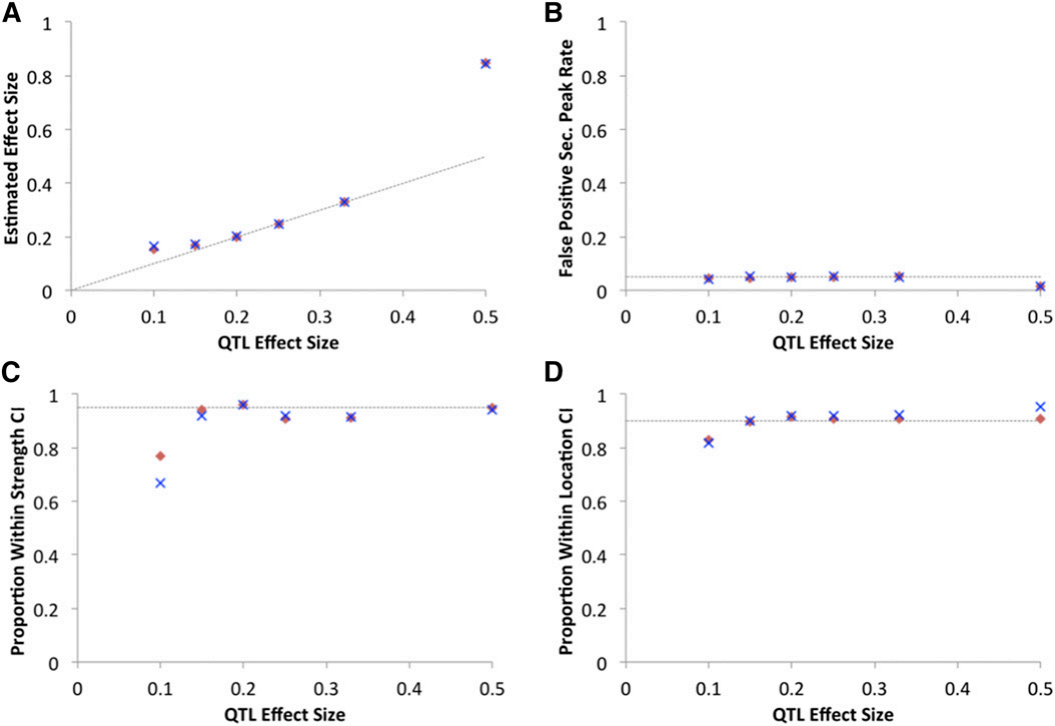
SIBSAM inferences largely conform to expectations. (A) For the same test simulations analyzed in Figure 7A, performance in estimating QTL effect size (the proportion of phenotypic variance explained). Accurate performance was obtained for intermediate strength QTL. Some overestimation of effect size was observed for the weakest QTL (due to a winner’s curse in which only the taller peaks are detected) and for the strongest QTL (due to saturation of the *a*_d_ statistic). (B) For a given primary peak, the probability of falsely inferring a secondary peak was near or below the expected 5% for all QTL strengths. (C) The proportion of replicates in which the SIBSAM effect size confidence interval contains the true value is typically close to the 95% expectation, but somewhat reduced for the weakest QTL simulated. (D) The proportion of replicates in which the SIBSAM genomic confidence interval contains the true simulated position was close to the expected proportion but again slightly reduced for the weakest QTL.

The estimation of QTL strength for significant peaks was quite accurate for intermediate strength QTL (15–33%) when the remaining phenotypic variance was random and normally distributed (Figure 8A). However, in other scenarios, the strength estimate could be overestimated. For a weaker QTL (*e.g.*, 10% in this example), there appears to be a “winner’s curse” in which only the test replicates giving the tallest peaks were deemed significant, and since these peaks are unusually high for a *s* = 10% QTL, their strength was typically overestimated. If strength estimates for nonsignificant peaks were included, there was no directional bias. The highest QTL strength (50%) showed upward bias, which may reflect a “saturation effect” of the *a*_d_ statistic. Here, peak heights were very close to 1 (individuals were well sorted into the extreme pools based on QTL genotype), which is the same outcome produced by a QTL with *s* > 50%. Upward strength bias was also observed if the remaining phenotypic variance was produced by other strong QTL, rather than normally distributed random variance. If a 20% QTL was accompanied by an unlinked 80% QTL (with no environmental/measurement variance), the median estimate of *s* was 24.2%. If a 20% QTL was accompanied by four unlinked QTL of equal strength, the median estimate of *s* was 31.4% (although power increased from 94 to 100% for both of these cases). In light of the recurrent bias in effect size estimation, the reported quantities are best viewed as rough estimates of QTL strength. Future methodological studies may explore alternative approaches to the estimation of QTL strength in a simulation framework.

Other aspects of SIBSAM inference performed largely as expected on the simulated data. Based on 10,000 null simulations, the false positive probability for detecting a QTL when none existed was 3.46%. In simulations that had a single true QTL, only ∼5% of significant primary peaks had a false positive secondary peak (in line with null expectations; Figure 8B). Thus, for a strong QTL associated with very high power (*e.g.*, *s* = 25%), there is an ∼95% probability of correctly inferring a single QTL and a 5% chance that a secondary QTL will be incorrectly suggested. For QTL strengths with adequate power, approximately the predicted proportion of loci fell within the provided confidence intervals for QTL strength and genomic position (Figure 8, C and D), with performance only declining for the weaker *s* = 10% case that was rarely detected for this scenario.

**Figure 9:**
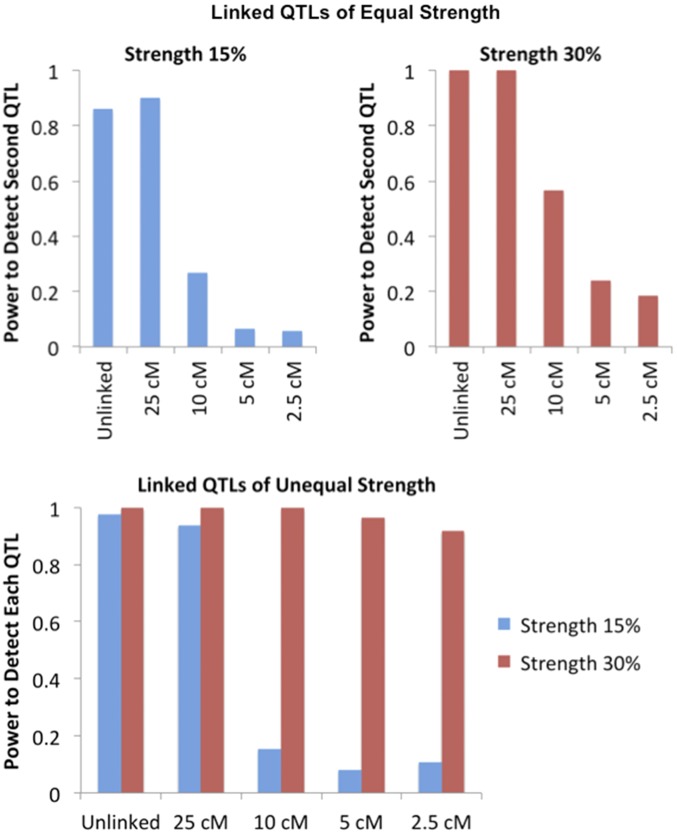
The detection power of SIBSAM in test simulations with two linked QTL. Top panels illustrate the power to detect the second of two linked QTL of equal magnitude, conditional on detecting the first. The bottom panel illustrates the power to detect either the weaker or the stronger of two linked QTL of unequal sizes.

Detection power was also examined for cases involving two linked QTL (of strength 15% and/or 30%) separated by various distances (2.5, 5, 10, and 25 cM). For QTL of equal strength, the 25-cM linkage had no adverse effect on QTL detection. Power was actually slightly higher in the case of two 15% QTL separated by 25 cM (relative to the unlinked case), even though 55% of these test replicates had one of the QTL as a secondary peak. Power to detect a second peak dropped significantly as the distance between QTL dropped to 10 and 5 cM (Figure 9). In the case where one QTL had *s* = 30% and the other had *s* = 15%, power remained high for the stronger QTL at all distances, but was low for weaker QTL at 10 cM or closer (Figure 9).

Power for a 15% QTL was notably higher when an unlinked 15 or 30% QTL was present, compared to the single QTL scenario in which all other phenotypic variation was due to normally distributed random variance (Figure 9 *vs.* Figure 7). Together with the multi-QTL results described above (in which power for a 20% QTL increased in the presence of an unlinked 80% QTL or else four other 20% QTL), these findings suggest that power to detect a QTL may be higher than indicated in Figure 7 when at least some of the remaining phenotypic variance comes from other QTL of appreciable strength, instead of random variance.

## Discussion

Mapping the genetic architecture of phenotypic trait differences remains a challenging but critical problem in the fields of genetics and evolutionary biology. Above, I have compared the behavior of bulk segregant analysis and introgression mapping, while assessing the experimental parameters that modulate their outcomes. I then offered a new simulation-based approach to BSA inference, geared toward systems like *Drosophila* in which hundreds or thousands (but not millions) of individuals can be examined, and in which BSA QTL signals may sometimes overlap each other.

A general principle of QTL mapping is that performance is enhanced by sampling a diverse range of recombinant genotypes. Thus, simulation results suggest that BSA and IM should both be more successful when more generations of interbreeding occur, when larger numbers of individuals are present in the mapping population, and when greater sequencing effort is employed. The importance of sampling at least a few tens of individuals in phenotypically selected pools is clear as well. These results suggest that the typical method of introgression mapping, in which small numbers of individuals are phenotypically selected every generation or two, is not advisable for mapping oligogenic traits (and is not ideal for monogenic traits either; Figure 4). Instead, if IM is used, larger numbers of phenotyped and retained individuals are desirable. However, based on the criteria employed here, BSA gave a more precise mapping signal than IM for every combination of experimental and QTL parameters examined. This finding may again relate to the principle of maintaining a diversity of recombination breakpoints, which is maximized by avoiding IM’s population bottlenecks associated with phenotypic selection during the intermediate generations of interbreeding.

The tradeoffs among BSA, IM, and other mapping approaches are complex and merit further attention. A compelling advantage of BSA is that the same experimental population may be used to map multiple trait differences (*e.g.*, once the adults have already reproduced, select for one trait in generation 12, another trait in generation 13, *etc*.). For the same set of experimental parameters as defined here, BSA actually requires less effort than IM during the experiment, since phenotyping must be performed only in the last generation. BSA does require the sequencing of two phenotypic pools (high and low), whereas IM requires just one phenotypic pool to be sequenced (note, however, that doubling IM depth does not allow it to match BSA’s performance; Figure S1). Because both parental strains’ genotypes are present across the genomes of mapping population individuals, BSA may be more influenced by the complexities of epistatic interactions. IM also results in the production of a genetically stable strain, which may prove useful in downstream experiments.

In the course of a BSA experiment, parental strain ancestry frequencies in the mapping population could deviate from 50%. The effects of genetic drift should be modest when the population size is vastly greater than the number of generations of interbreeding, and SIBSAM allows for drift’s occurrence. Although not modeled here, inadvertent laboratory selection could also shift mapping population ancestry frequencies. In general, such ancestry shifts should not lead to false positive QTL, because both phenotypic pools will be equally affected. If ancestry frequencies become extreme, the response of *a*_d_ to a QTL could be dampened, leading to reduced power and underestimation of QTL strength. This concern could be amplified for crosses between partially reproductively isolated species. But even for within-species mapping, it may be worthwhile to collect BSA sequence data before an excessive number of generations have elapsed. Genomic regions found to show ancestry shifts could be interesting in their own right, since they may contain drivers of laboratory adaptation, differential mating success, incompatibilities, or segregation distortion.

It is more challenging to compare BSA or IM against alternative mapping methods such as those involving individual genotyping (*e.g.*, Andolfatto *et al.* 2011) or the generation of recombinant inbred lines (*e.g.*, King *et al.* 2012). However, it may be worth evaluating the benefits of combining elements of BSA with these approaches. Following multiple generations in a large mapping population, offspring with extreme phenotypes could be individually genotyped. Or, the mapping population could be used to found a large number of recombinant inbred lines (RILs), with BSA and RIL mapping potentially integrated.

The mapping approach and method described here requires a moderate investment of researcher time and funding and delivers a range of QTL inferences. While useful in its current form, SIBSAM may also motivate future simulation-based mapping methods. Although motivated by *Drosophila* QTL mapping, this approach may prove broadly useful for nonmodel insects and other smaller organisms with short generation times.
